# Transcription Inhibition as a Therapeutic Target for Cancer

**DOI:** 10.3390/cancers3044170

**Published:** 2011-11-23

**Authors:** Christine M. Stellrecht, Lisa S. Chen

**Affiliations:** Department of Experimental Therapeutics, The University of Texas MD Anderson Cancer Center, Unit 1950, PO Box 301429, Houston, TX 77230-1429, USA; E-Mail: lschen@mdanderson.org

**Keywords:** transcription inhibitors, oncogene addiction, gene expression, ribonucleoside analogs, cyclin–dependent kinase inhibitors

## Abstract

During tumorigenesis the transformed cells lose their normal growth control mechanisms and become dependent on oncogenes' products and pathways for survival. Treatments tailored to block the expression or function of transforming genes have shown efficacy in eliminating neoplastic cells. The mRNAs of many oncogenes, as well as regulators of other key processes such as cell proliferation, angiogenesis, and apoptosis, typically have shorter half-lives. Agents that impede mRNA synthesis are expected to selectively hinder the expression of these genes and, therefore, be detrimental to neoplastic cells that are physiologically dependent on them. In addition to exploiting the tumor cells' dependency on short-lived transcripts, RNA-directed agents also take advantage of the differential sensitivity between transformed and non-transformed cells, as the cytotoxic effects of inhibiting RNA synthesis have not been seen in non-transformed cells. The abrogation of the formation of oncotranscripts provides a new concept in cancer therapeutics and numerous agents have been developed which are able to target transcription. The focus of this review is to give an overview of transcription and the different inhibitory strategies that target various aspects of the transcriptional process.

## 1. Introduction

The genesis and survival of many tumors are uniquely dependent upon the activity of oncogenes that confer a gain of function. This dependency/addiction leads to growth, maintenance, and survival advantages for the clonally transformed cells. Since the first known oncogene, *src*, was isolated as the transforming genetic material of the Rous sarcoma virus, more than 100 oncogenes have been found to be activated during carcinogenesis and tumor progression. These include oncogenes activated by altered regulation due to retroviral integration and chromosomal translocation, such as *MYC*, as well as those constitutively activated by amplification, gene rearrangement, and truncating and point mutations. The latter group includes receptor tyrosine kinases (the epidermal growth factor receptor *HER-2/NEU* and the hepatocyte growth factor receptor *MET*), cytoplasmic kinases (*SRC* and *BCR/ABL*, the rearranged gene product of the Philadelphia chromosome), and GTPase signal transducers (*RAS*). In addition, similar alterations or over-expression of cell cycle regulators (cyclin D1) and anti-apoptotic molecules (*BCL2* family members) have been established as playing a vital role in carcinogenesis or survival [1,2].

During tumorigenesis, the transformed cells lose their normal growth control mechanisms and become dependent on the oncogene(s) products for survival [1]. Treatments tailored to block the expression or function of transforming genes have shown efficacy in eliminating the neoplastic cells. This could be achieved by inhibiting synthesis of the oncotranscript or oncoproteins. Because the first step in oncogene expression is transcription, agents that impede mRNA synthesis offer an effective approach for abrogating their expression [3,4]. Although these agents globally affect RNA synthesis, their actions are selective for transcripts, which have more rapid turnover. Typically, short-lived transcripts are encoded by oncogenes, as well as regulators of other key processes such as cell proliferation, angiogenesis, and cell survival [5-7].

Thus, treatments that target RNA synthesis are expected to selectively hinder the expression of short-lived transcripts and, therefore, be detrimental to neoplastic cells that are physiologically dependent on these genes. This type of approach provides a new concept in cancer therapeutics and is the focus of this review.

## 2. The mRNA Synthesis Process

### 2.1. Formation of the Pre-Initiation Complex

**Figure 1.**
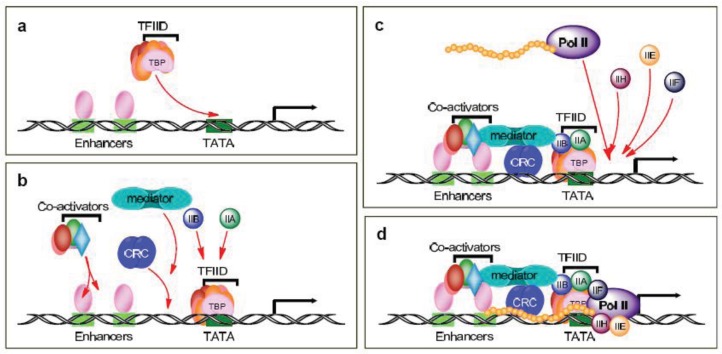
Schematic diagram of the formation of the pre-initiation complex. (**a**) Recruitment of transcription factor IID (TFIID). (**b**) Mobilization of mediator, chromatin remodeling complex (CRC), and other co-activators. (**c**) Recruitment of Pol II and the transcription factors IIE, IIF, and IIH. (**d**) The completed pre-initiation complex.

Gene expression is a highly regulated multi-step process in which each step provides a potential target for drug development. The first steps entail the formation of the pre-initiation complex and involve the binding of various transcription factors to DNA and the recruitment of RNA polymerase II (Pol II) to the transcriptional start site; the complexities of these activities are still not fully appreciated. In the traditional scenario, sequence-specific activators bind distal and proximal enhancer elements of a gene and recruit transcription factor IID (TFIID) to the core promoter (Figure 1a). TFIID is a large multisubunit complex that includes the TATA-binding protein (TBP) and TBP-associated factors (TAFs). In actuality, there are multiple modes of promoter recognition, due in part to the use of other basal elements besides the TATA box as well as tissue-specific TAFs and other factors related to TBP.Thus, there are multiple types of pre-initiation complexes, with unique combinations of TBP and TAFs, which further support gene- and tissue-specific regulation (reviewed in [8,9]).

Following the activators and/or TFIID recruitment, in the traditional scenario, the chromatin remodeling complexes (CRC), the Mediator and other coactivators, as well as TFIIA and TFIIB, are recruited to the pre-initiation complex (Figure 1b). The Mediator bridges the interactions between the activators and the basal initiation machinery and, for the most part, is required for transcription activation. It is composed of at least 24 subunits and the varied subunit composition of the Mediator also allows for cell-type- and promoter-specific transcription. TFIIA is composed of two subunits and behaves as a coactivator and counteracts the repressive effects of negative cofactors. TFIIB is a single subunit that stabilizes TFIID-promoter binding and provides accurate start site selection and direction of transcription.

### 2.2. Transcription Initiation

**Figure 2.**
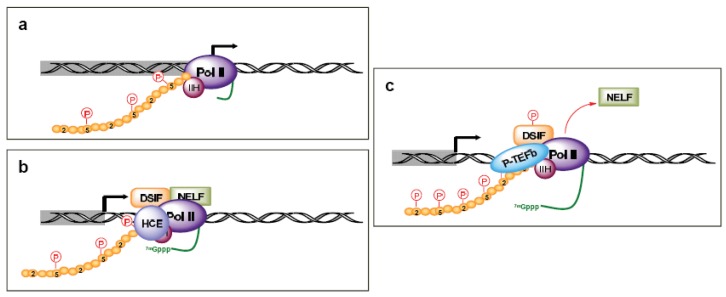
Schematic diagram of promoter clearance and the transition to an elongation complex. (**a**) Promoter clearance, with the phosphorylation of serines at position 5 of the C-terminal domain (CTD). (**b**) Recruitment of 5,6-dichloro-1-β-d-ribofuranosylbenzimidazole (DRB) sensitivity-inducing factor (DSIF) and negative elongation factor (NELF) to induce pausing of Pol II and the binding human capping enzymes (HCE) to generate a 5′ m7G cap on nascent transcripts. (**c**) Recruitment of P-TEFb with the phosphorylation of serines at position 2 in the CTD, followed by productive elongation. Phosphorylation of DSIF and Pol II on Ser2 and 5 in the heptapeptide repeats of the CTD are depicted by red letters in red circles. The nascent transcript is depicted as a green line.

The TFIID/TFIIA/TFIIB complex is then able to sequentially recruit TFIIE, TFIIF, Pol II, and TFIIH to the pre-initiation complex (Figures 1c and 1d). TFIIE is composed of two subunits and is essential for promoter melting. Additionally, it helps recruit and stimulate TFIIH. The three subunits of TFIIF may help keep the DNA tightly wrapped around the pre-initiation complex as well as enhance the affinity of Pol II for the promoter complex. Pol II contains 12 subunits, the largest of which is RPB1. The C-terminal domain (CTD) of the human RPB1 contains 52 heptapeptide repeats (YSPTSPS) that are essential for Pol II activity. TFIIH plays a crucial role in both transcription initiation and promoter clearance. It is composed of 10 subunits, seven of which (XPD, XPB, p62, p52, p44, p34, and TTDA) form the core complex. XPB and XPD have helicase and ATPase activities, which are necessary for promoter opening as well as transcription-coupled DNA repair. The cyclin-activating kinase-subcomplex (cyclin–dependent kinase (CDK) 7, MAT1, and cyclin H) is linked to the core via the XPD protein. CDK7/cyclin H can directly phosphorylate transcription factors and the CTDs Ser5 and -7 in the heptapeptide repeats (Figure 2a), but there are conflicting reports of the role of this phosphorylation on Pol II activity and promoter escape [10-15], as additional factors likely complicate the issue [12,16].

### 2.3. Transcription Elongation

After the first 25–30 nucleotides are transcribed, there is a transition between transcription initiation and elongation [17-20]. This is marked by several events, including the enhanced stability of the DNA-RNA-transcription machinery, promoter release, binding of elongation factors, and the hyperphosphorylation of the Pol II CTD. This process also coordinates the first pre-mRNA processing reaction by arresting Pol II at a “checkpoint”. This Pol II arrest is accomplished through TFIIH kinase activity promoting the recruitment of 5,6-dichloro-1-β-d-ribofuranosylbenzimidazole (DRB) sensitivity-inducing factor (DSIF) and negative elongation factor (NELF) (Figure 2b). The pause in Pol II activity allows for the addition of the 7-methyl G5′ppp5′N cap structure to the RNA transcript, which aids in stability, splicing, nuclear export, and translation [20]. The human capping enzymes (HCE) associate physically with the Ser5 phosphorylated CTD. Release from the checkpoint involves the kinase activity of P-TEFb, which includes Cdk9 coupled with cyclin T. P-TEFb is recruited by the capping enzymes and phosphorylates DSIF (Figure 2c). This leads to the release of NELF and relieves Pol II pausing, though there is gene specificity in this process as well [21]. Additionally, P-TEFb phosphorylates the Pol II CTD Ser2, which coordinates both transcription elongation and termination. CTD Ser2 phosphorylation recruits elongation factors, such as CRC, as well as splicing complexes [18].

### 2.4. Transcription Termination

Transcription termination requires both cleavage and polyadenylation of the RNA transcripts as they extended beyond the site of polyadenylation [22]. The 3′-end processing complex contains over a dozen proteins, several of which bind the CTD of Pol II. Most notable is poly(A) polymerase (PAP), but also includes cleavage and polyadenylation specificity factor (CPSF), cleavage stimulation factor (CstF), cleavage factor I (CFIm), cleavage factor II (CFIIm), poly(A)-binding protein (PABP), and symplekin. Interestingly, some of these factors are recruited during transcription initiation and interact with transcription factors. CPSF contains five subunits and all are required for efficient cleavage and polyadenylation. Evidence indicates that CPSF-73 is the cleavage endoribonuclease. Many of the other factors are involved with RNA binding, polyadenylation site recognition, and scaffolding. The processing 3′-end is crucial not only because it is coupled to the transcription and splicing machineries but also because it promotes stability of mRNAs and their transport from the nucleus to the cytoplasm, as well as enhances protein translation.

## 3. Therapeutic Inhibitors of mRNA Synthesis

The intricacy of RNA transcription provides numerous potential drugable targets. Therapeutic interventions have been developed for many of the steps in transcript synthesis; however, fewer than a dozen of these interventions have reached the clinic (Table 1). Therapeutically, RNA-directed agents provide some important advantages as a treatment option. First, evidence indicates that they do not pose a general cytotoxicity, as is seen with DNA-directed therapies. Inhibition of Pol II in untransformed cells such as fibroblasts [23], normal lymphocytes [24-27], and mammary epithelial cells (unpublished) does not readily induce apoptosis. Instead, it has been demonstrated that in untransformed fibroblast and epithelial cells, transcription inhibitors induce a reversible growth arrest. This differential sensitivity between transformed and non-transformed cells appears to be due to oncogene addiction. It is this differential sensitivity that first gave the implication of pursuing the strategy of therapeutically exploiting transcription inhibition. Specifically, the response to transcription inhibition in non-transformed fibroblasts engineered to express an inducible *MYC* construct dramatically changed from growth inhibition to apoptosis induction by 12 hr of stimulation of *MYC* expression [23]. Moreover, the hypothesis for the mechanism of the differential sensitivity has been strengthened by numerous proof-of-principle studies that have demonstrated the depletion of oncogenic pathways in various therapeutic models [24,28-41].

Though any tumor type that is dependent on a short-lived factor(s) would benefit from RNA-directed therapy, currently there is added focus on clinical trials treating indolent malignancies with these agents. Traditionally, indolent diseases have been difficult to treat because of their low proliferative index, which deters the effectiveness of DNA-directed therapies. Additionally, the presence of multiple anti-apoptotic signals promotes therapeutic resistance. Many indolent cancer cells are highly dependent on anti-apoptotic signals, which are short-lived and thus easily amendable to transcription inhibition.

### 3.1. Inhibitors of RNA Chain Synthesis

#### Actinomycin D

**Figure 3.**
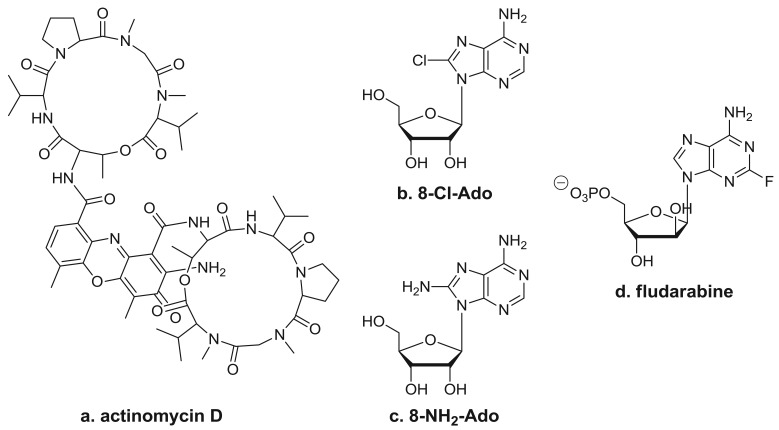
Inhibitors of RNA chain synthesis. Structures of (**a**) actinomycin D, (**b**) 8-Cl-Ado, (**c**) 8-NH_2_-Ado, and (**d**) fludarabine.

One well-known RNA synthesis inhibitor that is used in the clinic is actinomycin D (Figure 3a). It works by intercalating into DNA, preventing topoisomerase I from releasing covalently linked DNA, stabilizing the nicked intermediate in the cleavage and resealing reaction. In the clinic, this agent is used for the treatment of several tumor types, which include Wilms' tumor, rhabdomyosarcoma, Ewing's sarcoma, and trophoblastic neoplasias. *In vitro* studies have demonstrated that long exposure to low doses (0.01–0.25 μg/mL) of actinomycin D or shorter exposure to higher doses (1–2 μg/mL) inhibits the synthesis of all RNA species. Limited pharmacokinetic studies performed during therapy suggest that repeated doses of 15 μg/kg may reach the level needed for RNA synthesis inhibition [42]; however, studies on the *in vivo* effects of actinomycin D on gene expression in clinical samples are lacking [8,9]. Because actinomycin D also is DNA-directed, it does produce side affects typical of these types of agents.

#### α-Amanitin

The mushroom toxin α-amanitin is a cyclic peptide of eight amino acids. It acts by binding directly to Pol II, putting constraints on its mobility, hence slowing down the rate of RNA synthesis [43]. Its irreversible actions, as well as free radical reactions, contribute to severe alpha amanitin hepatotoxicity [44]; thus precludes its use in cancer therapy.

### 3.2. Premature Transcription Chain Terminators

#### 8-Cl-Ado and 8-NH_2_-Ado

By virtue of their resemblance to ATP, which is a precursor of RNA synthesis, it was hypothesized that C8-substituted adenosine analogs such as 8-chloroadenosine (8-Cl-Ado) and 8-aminoadenosine (8-NH_2_-Ado) (Figures 3b and 3c) would incorporate into the RNA body and poly(A) tail and inhibit further transcript synthesis [45]. The 2′-OH residue in the sugar moiety makes these congeners RNA-directed without having any direct effect on DNA synthesis [45-47]. The cytotoxicity of 8-Cl-Ado has been shown to be contingent on its intracellular phosphorylation to 8-Cl-AMP by adenosine kinase [45], which is followed by the formation of 8-Cl-ATP. Due to adenosine kinase's high specific activity and substantial substrate specificity for 8-Cl-Ado, 8-Cl-ATP accumulates to near-mM levels [41,45,48]. Moreover, 8-NH_2_-ATP accumulates to as high as ∼6 mM [28]. The accumulation of these analogs' cellular metabolites parallels a progressive decline in the endogenous ATP pool. The favorable ratio of C8-substituted-ATP to ATP facilitates increased insertion of the analog in RNA, leading to premature transcript termination [74,49]. Of the various types of transcripts, mRNA synthesis is inhibited the most due to the preferential incorporation of these ATP analogs by Pol II into the body of the mRNA [47] and the likely detrimental effects on poly(A) polymerase and polyadenylation [50].

Our group and others have shown that the ribonucleoside analogs 8-Cl-Ado and/or 8-NH_2_-Ado inhibit RNA synthesis in primary chronic lymphocytic leukemia (CLL) cells [24,28], as well as in multiple myeloma (MM) [45,47], mantle cell leukemia [25,51], glioma [52], and breast [48] cancer cell lines. Moreover, 8-Cl-Ado and/or 8-NH_2_-Ado have been shown to be tumoricidal to these cells as well as other malignant cells [24,28,39,45,48,52-60]. The tumoricidal actions of these RNA-directed nucleoside analogs have been shown to be the result of depletion of the expression of oncogenes that the malignant cells are dependent on for survival [24,28,39]. In contrast, in normal lymphocytes [24,25] and in the non-malignant MCF-10A mammary epithelial cell line (unpublished results), 8-Cl-Ado and/or 8-NH_2_-Ado were not cytotoxic, presumably due to the lack of oncogene addition in non-transformed cells.

The differential sensitivity of transformed and non-transformed cells to the tumoricidal activity of 8-Cl-Ado and 8-NH_2_-Ado is expected to translate into a favorable therapeutic index in the clinic. Consistent with this expectation, mouse toxicological assessment of 8-Cl-Ado showed no toxicity at doses above those anticipated to be tumoricidal (unpublished data). In cellular pharmacological analyses performed on mouse peripheral blood mononuclear cells after intravenous administration of 50 and 100 mg/kg 8-Cl-Ado, 8-Cl-ATP accumulated within 1 hr to ∼350 and ∼1150 μM, respectively [61]. These levels of accumulation were much higher than the tumoricidal levels that accumulated in cultured cells treated with 10 μM 8-Cl-Ado [45,48]. In the toxicology studies, in which 150, 250, and 350 mg/kg 8-Cl-Ado was administered intravenously daily for 5 days, only the 250 and 350 mg/kg/daily dose showed signs of a persistent nephrotoxicity, while no other pathologic or hematologic toxicologically important alterations were observed. More importantly, 8-Cl-Ado is currently undergoing phase I dose-escalating clinical trial in previously treated CLL patients at The University of Texas MD Anderson Cancer Center. The doses administered thus far are 45, 67.5, and 100 mg/m^2^/d for 5 days, and there have been no signs of toxicity. One of the patients receiving 100 mg/m^2^/d was assessed for 8-Cl-ATP accumulation in their leukemia cells, and the levels were measured to reach 100 μM by day 5 [62].

#### Fludarabine

Fludarabine (Figure 3d) is a clinically approved nucleoside analog that targets both DNA- and RNA-directed processes. Therefore, its role in the inhibition of specific transcripts is difficult to study in replicating cell line systems. Nonetheless, its mechanism of action may be exclusively RNA-directed in indolent malignancies such as CLL [63]. Incorporation of this analog into nascent mRNAs also results in the premature termination of the transcripts and depletion of proteins required for cell survival.

### 3.3. Inhibitors of Pre-mRNA Cap Formation

#### Neplanocin A and 3-deazaneplanocin A

Adenosine analogs, such as neplanocin A and 3-deazaneplanocin A, have been used to inhibit methylation of RNA, including the guanine-7-methylation of the cap structure. These agents affect the biosynthesis of S-adenosylhomocysteine, raising its levels, which in turn inhibits many methyltransferases [64,65]; thus, they also produce epigenetic effects [66]. In culture, these analogs exhibit antitumor properties, and neplanocin A has been shown to selectively decrease the stability of the *Myc* oncogene in murine erythroleukemia cells but not housekeeping genes [67].

#### 3.4. Transcription Elongation Inhibitors

**Figure 4.**
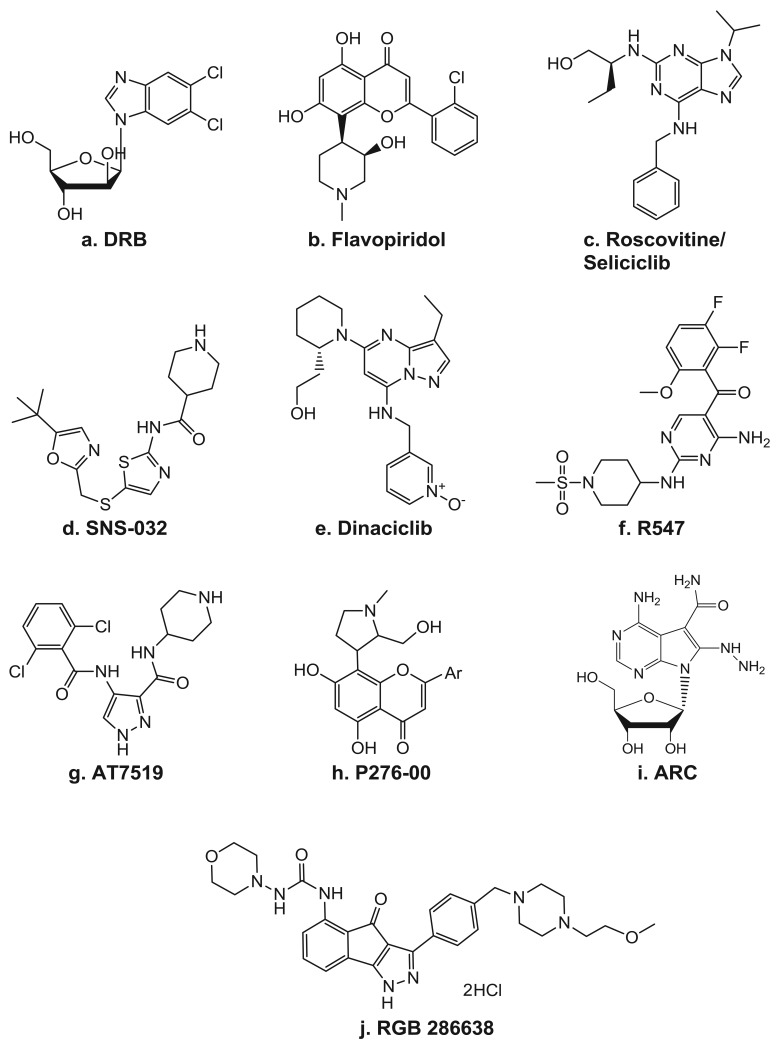
CDK inhibitors. Structures of (**a**) DRB, (**b**) flavopiridol, (**c**) seliciclib (**d**) SNS-032, (**e**) dinaciclib, (**f**) R547, (**g**) AT8319, (**h**) P276-00, (**i**) ARC, and (**j**) RGB 286638.

Because CDKs were originally discovered as key regulators of cell cycle progression, inhibitors of CDKs were initially studied for their ability to arrest the cell cycle. Now, as the role of CDKs in transcription has emerged, numerous inhibitors with higher specificity to the TFIIH component, CDK7, and even more so to P-TEFb's CDK9, are being developed and brought into the clinic (Table 2). In fact, the first identified small molecule inhibitor of transcription was the ATP analog DRB (Figure 4a), which is now known to be an inhibitor of P-TEFb [68]. As a CDK inhibitor, DRB has activity highly specific for CDK9 and has proven to be a very useful tool for delineation of the various transcription elongation factors. Though the effects of many of the CDK inhibitors often involve alterations of factors involved in the cell cycle, there have also been clear indications of their ability to target transcription.

#### Flavopiridol/alvocidib

The pan-CDK inhibitor flavopiridol (Figure 4b) has been shown to impede transcription by inhibiting CDK9 [69]. It is a semi-synthetic flavone derived from a plant indigenous to India known as *Dysoxylum binectarieferum* [70]. Flavopiridol was first brought into clinical trials on the basis of its preclinical activities of inducing cell cycle arrest and tumor growth inhibition [71]. In cycling cells *in vitro*, it blocks cell cycle progression at the G1 and G2 phases [72]. In addition to being a CDK inhibitor, it was also reported to have activity, albeit lower, against other kinases, including PKC, pp60 Src, EGFR, and Erk-1 [73].

Paradoxically, the first strong indication of the clinical activity of flavopiridol was not observed in trials against proliferating tumor types but instead in a phase I clinical study in refractory, high-risk CLL [87]. In fact, CLL is characterized by the accumulation of nonproliferating, mature B cells, most of which are in G_0_/G_1_ phase caused by failed programmed cell death [88]. The problem in the first clinical trials appears to be that flavopiridol binds to human plasma proteins, reducing its free concentration and, thus, making continuous intravenous infusion dosing ineffective. In the phase I CLL trial, flavopiridol was administered first as a 30-min intravenous bolus followed by a 4 hr continuous intravenous infusion. This strategy achieved the serum concentrations necessary to induce *in vivo* apoptosis and produced a 40% partial response rate [89]. In fact, the dose-limiting toxicity of flavopiridol is the direct result of the *in vivo* apoptosis, as it produces an acute tumor lysis syndrome that requires careful monitoring during treatment [90]. Furthermore, flavopiridol-induced apoptosis in CLL has been correlated with the loss of Pol II CTD phosphorylation on Ser2 and 5, leading to inhibition of *MCL-1* and *XIAP* transcription, followed by depletion of their respective proteins [31,91]. Similar results have been noted in acute myeloid leukemia [92] and multiple myeloma [32] cells treated with flavopiridol. Impressively, in a phase II trial, a 53% overall response rate was obtained [93]. Additionally, an overall response rate of 82% and a complete response rate of 50% have been seen in a phase I combination study of flavopiridol with fludarabine and rituximab in patients with mantle-cell lymphoma, indolent B-cell non-Hodgkin's lymphomas, or CLL [94]. Currently, it is being used in several other clinical trials as a single agent or in combination studies. Most of these trials are concentrated on B-cell malignancies, though there are some in solid tumors as well.

As mentioned above, the dose-limiting toxicity of flavopiridol is due to acute tumor lysis syndrome. In diseases such as CLL, frequently the tumor burden is extremely large. The immense and rapid killing of this large population of tumor cells results in a rapid release of break-down products from the dying tumor cells. This may induce metabolic complications such as hyperuricemia which leads to the risk of acute renal failure. Because of this, treatment of CLL patients with transcription inhibitors requires careful monitoring during treatment [90].

*Seliciclib/CYC-202/Roscovitine* (Figure 4c) is a 2,6,9-substituted purine analog that competes with ATP for binding to the active site on CDKs. It has potent *in vitro* activity against CDK2/cyclin E, CDK7/cyclin H, and CDK9/cyclin T, while CDK4, -6, -8 and most other kinases are not affected [95]. Seliciclib arrests the cell cycle in the G_1_, S, or G_2_/M phase, depending on the concentration, the length of treatment, and the cell line used. It can induce cell death in all phases of the cell cycle and is active against a wide range of tumor cell lines [34,36,96-98]. The seliciclib-induced cell cycle alteration is due to both direct and indirect effects of its actions. The majority of the indirect mechanisms stem from changes in the gene expression profile, which include depletion of the mitotic regulators aurora A and B, polo-like kinase, WEE1, and CDC25C [98]. Additionally, like flavopiridol, seliciclib has been reported to induce the depletion of several anti-apoptotic factors, such as MCL-1, XIAP, and survivin [26,34,36,97]. In CLL cells, Hallaert et al. [99] demonstrated that the depletion of MCL-1 induces a Noxa-dependent BIM displacement and liberation, which leads to BAX activation. In addition to its ability to directly affect transcription by inhibiting CDK7 and CDK9 activity, treatment with seliciclib also leads to the depletion of several general transcription factors, such as TFIIB and TFIID [26]. Seliciclib was the first orally bioavailable CDK inhibitor in clinical trials. It has shown promising results in several phase 1 and 2 clinical trials as a single agent against B-cell malignancies, non-small cell lung cancer, and nasopharyngeal cancer [100]. Currently, seliciclib is also being evaluated in several combination trials in advanced solid tumors.

*SNS-032/BMS-387032* (Figure 4d) was first known as a strong and selective inhibitor of CDK2 as compared to CDK1 and 4 as well as a panel of unrelated kinases [80]. Later, it was shown to also be a potent inhibitor of CDK7 and 9 [77]. In preclinical studies, SNS-032 showed efficacy against primary CLL cells [101], as well as against acute myeloid leukemia cell lines [102]. The *in vivo* activity of SNS-032 was confirmed in various animal models [80], including the P388 murine leukemia, the Br-CycE murine breast carcinoma, the A2780 human ovarian carcinoma, and the RPMI-8226 human myeloma xenograft tumor models. Preclinical studies demonstrated that the cytotoxic activity of SNS-032 correlates with its transcriptional inhibition and elimination of *MCL-1* expression in primary CLL cells and the majority of mantle cell leukemia cell lines [101,103].

On the basis of its kinase selectivity, its preclinical activity, and its moderately low plasma protein binding (63%), SNS-032 was chosen for clinical development. In a phase I trial in patients with CLL and multiple myeloma, SNS-032 showed modest clinical activity [104]. Biomarker analysis on the patients' leukemia cells during treatment demonstrated a reduction in Pol II CTD Ser2 phosphorylation and diminished *MCL-1* expression during the 6 hr infusion period, which rebounded post-infusion, suggesting a longer infusion time may be warranted.

*Dinaciclib/SCH 727965* (Figure 4e) is a pyrazolo[1,5-a]pyrimidine that was identified on the basis of its efficacy and tolerability in xenograft models [81,82]. Studies in osteosarcoma cells demonstrated a dinaciclib CDK2-mediated cytotoxicity, which was also associated with depletion of *MCL-1* and *BCL-XL* transcripts [105]. Dinaciclib is currently in multiple phase 1 and 2 clinical trials in various solid tumors and hematological malignancies.

*AT8319* (Figure 4g) is a pan-CDK inhibitor currently being tested in clinical trials in patients with multiple myeloma, advanced solid tumors, and refractory non-Hodgkin's lymphoma. In preclinical analyses, it was shown to be able to inhibit Pol II CTD Ser2 phosphorylation and global RNA synthesis at nM concentrations, which was associated with decreased expression levels of *MCL-1* and *XIAP* [84,106].

#### Other Cdk inhibitors

R547 (Figure 4f) is a diaminopyrimidine that is highly specific against CDK 1, 2, 4, 7, and 9 [83] and has recently completed testing in a phase 1 clinical trial. Phase II trials in advanced solid tumors and hematologic malignancies are being planned. P276-00 (Figure 4h) is a rohitukine derivative. It was found to be highly selective for cancer cells as compared with normal fibroblast cells, as nM quantities readily inhibited the growth of several colon cancer as well as osteosarcoma, leukemia, breast, cervical, prostate, bladder, and lung cancer cell lines but not of two different normal lung fibroblast cell lines [85]. In multiple myeloma cells, P276-00 cytotoxicity also correlated with inhibition of Pol II CTD Ser2 phosphorylation, decreased growth, and inhibition of survival proteins such as MCL-1 and cyclin-D_1_ [107]. P276-00 is currently in clinical trials in combinational studies.

A clinical trial of RGB 286638 (Figure 4j) will be recruiting patients with hematological malignancies. In myeloma cells, while RGB 286638 clearly depleted Pol II CTD Ser2 phosphorylation, cytotoxicity was associated with both CDK-dependent and -independent inhibition [86]. The compound ARC [4-amino-6-hydrazino-7-β-D-ribofuranosyl-7H-pyrrolo(2,3-d)-pyrimidine-5-carboxamide; Figure 4(i)] has been shown to induce apoptosis in several types of cancer cells due to its depletion of anti-apoptotic proteins such as MCL-1 and survivin [40,41,108-110]. Using purified P-TEFb in an *in vitro* kinase assay on a recombinant Pol II CTD, ARC was shown to inhibit this kinase [41]. Moreover, there is evidence that the apoptosis is selective for transformed cells, as SV40-transformed human fetal lung fibroblasts underwent extensive apoptosis within 24 hr with ARC, whereas the wild-type fibroblasts were not susceptible to ARC-mediated cell killing, even at higher concentrations of ARC [41].

### 3.5. Polyadenylation Inhibitors

Transcript levels are held in balance by the rate of their synthesis versus their degradation. Although several aspects, such as the sequence of a transcript, the levels of a transcript and protein, and exogenous factors such as hormones, regulate mRNA stability, polyadenylation of mRNA has been established as a major determinant of transcript half-life and translational efficiency [111,112]. Increased message degradation may result from truncated poly(A)-tail synthesis. Hence, in addition to transcription, polyadenylation serves as a potential target for manipulations. Several ATP analogs, including 8-bromo-ATP, 8-Cl-ATP, 8-NH_2_-ATP, 8-azido-ATP, and cordycepin have been demonstrated to block polyadenylation in a yeast model system. The analogs inhibited polyadenylation via one of two modes of action: the direct incorporation and subsequent termination of poly(A) tail synthesis or the reduction of the poly(A)-tail length [50]. All of these agents have been shown to be effective against cancer cell lines. Additionally, both 8-Cl-Ado and cordycepin have been shown to be cytotoxic to myeloma cells, in part by depleting *MET* transcripts [30,39].

## 4. Conclusions

Historically, chemotherapeutic agents have been DNA-directed to affect DNA replication and repair. The present concept deals with RNA-directed strategies that become tumor-specific in a context-dependent manner. Proof-of-concept preclinical studies further provide encouragement for the development of drugs that block steps in the mRNA synthesis process. In addition, RNA-directed agents are also proving to be effective against tumors that are indolent and thus not amenable to DNA-directed therapies.

## Figures and Tables

**Table 1.** RNA-directed agents in clinical trials or in use as standard treatments.

**Compound**	**Class**	**Inhibitory Action**	**Type/Site of Cancer**
8-Cl-Ado	nucleoside analog	RNA chain synthesis	CLL*
actinomycin D	antibiotic	RNA chain synthesis	Wilms' tumor, Ewing's sarcoma, trophoblastic neoplasias
AT8319M	pyrazole amide	transcription elongation	MM, lymphoma, solid tumor
cordycepin	nucleoside analog	transcript polyadenylation	CML, ALL
dinaciclib/SCH-727965	pyrazolopyrimidine	transcription elongation	MM, plasma cell neoplasia, melanoma
flavopiridol/alvocidib	flavone	transcription elongation	MM, CLL, AML, lymphoma, germ cell tumor, melanoma
fludarabine	nucleoside analog	RNA chain synthesis	hematological malignancies
P276-00	flavone	transcription elongation	MM, breast, pancreas, MCL, HNSCC, melanoma
R547	diaminopyrimidine	transcription elongation	solid tumors
RGB-286638	pyrazole amide	transcription elongation	hematological malignancies
Roscovitine/Seliciclib	purine derivative	transcription elongation	breast, solid tumors
SNS-032	aminothiazole	transcription elongation	B-lymphoid malignancies, CLL, MCL, MM, solid tumors

* acute lymphocytic leukemia (ALL); chronic myelogenous leukemia (CML); chronic lymphocytic leukemia (CLL); head and neck squamous cell carcinoma (HNSCC); mantle cell lymphoma (MCL); multiple myeloma (MM).

**Table 2.** The IC_50_ for CDK inhibition, as measured by *in vitro* kinase assays.

**Inhibitor**	**[Ref.]**	**Cdk1 cyclin B**	**Cdk2 cyclin A**	**Cdk2 cyclin E**	**Cdk4 cyclin D**	**Cdk5 p35**	**Cdk6 cyclin D**	**Cdk7 cyclin H**	**Cdk9 cyclin T**
**DRB**	[74]	17,000	>10^4^	>10^4^	>10^4^			>10^4^	340
**Flavopiridol**	[75-77]	41	100	170	65	∼100	∼100	∼300	6
**Seliciclib**	[34,77-79]	650	700	100	>10^5^	160	>10^5^	360	600
**SNS-032**	[77,80]	480	38	48	925	340	>1000	62	4
**Dinaciclib**	[81,82]	3	1	NA	10	1	NA	70	4
**R547**	[83]	0.2	0.1	0.4	1	0.1	4	171	13
**AT7519**	[84]	190	44	510	67	13	170	2400	<10
**P-276-00**	[85]	79	224	2,540	63	NA	396	2870	20
**RGB 286638**	[86]	2	NA	3	4	5	55	44	1
